# The effect of previous SARS-CoV-2 infection on systemic immune responses in individuals with tuberculosis

**DOI:** 10.3389/fimmu.2024.1357360

**Published:** 2024-06-27

**Authors:** Mariana S. Xavier, Mariana Araujo-Pereira, Quezia M. de Oliveira, Flavia M. Sant’Anna, Felipe M. Ridolfi, Alice M. S. de Andrade, Marina C. Figueiredo, Timothy R. Sterling, Bhavna G. Gordhan, Bavesh D. Kana, Bruno B. Andrade, Valeria C. Rolla, Adriano Gomes-Silva

**Affiliations:** ^1^ Pós-graduação em Pesquisa Clínica em Doenças Infecciosas, Instituto Nacional de Infectologia Evandro Chagas, FIOCRUZ, Rio de Janeiro, Brazil; ^2^ Laboratório de Pesquisa Clínica e Translacional, Instituto Gonçalo Moniz, FIOCRUZ, Bahia, Brazil; ^3^ Curso de Medicina, Faculdade ZARNS, Bahia, Brazil; ^4^ Multinational Organization Network Sponsoring Translational and Epidemiological Research Initiative, Bahia, Brazil; ^5^ Laboratório de Pesquisa Clínica em Micobacterioses, Instituto Nacional de Infectologia Evandro Chagas, FIOCRUZ, Rio de Janeiro, Brazil; ^6^ Vanderbilt University Medical Center, Department of Medicine, Division of Infectious Diseases, Nashville, TN, United States; ^7^ Department of Science and Innovation/National Research Foundation Centre of Excellence for Biomedical Tuberculosis Research, School of Pathology, Faculty of Health Sciences, University of the Witwatersrand and the National Health Laboratory Service, Johannesburg, South Africa; ^8^ Laboratório Interdisciplinar de Pesquisas Médicas, Instituto Oswaldo Cruz, FIOCRUZ, Rio de Janeiro, Brazil

**Keywords:** tuberculosis, SARS-CoV-2, immune response, previous infection, disease severity, immunomodulation

## Abstract

**Background:**

The impact of previous SARS-CoV-2 infection on the systemic immune response during tuberculosis (TB) disease has not been explored.

**Methods:**

An observational, cross-sectional cohort was established to evaluate the systemic immune response in persons with pulmonary tuberculosis with or without previous SARS-CoV-2 infection. Those participants were recruited in an outpatient referral clinic in Rio de Janeiro, Brazil. TB was defined as a positive Xpert-MTB/RIF Ultra and/or a positive culture of *Mycobacterium tuberculosis* from sputum. Stored plasma was used to perform specific serology to identify previous SARS-CoV-2 infection (TB/Prex-SCoV-2 group) and confirm the non- infection of the tuberculosis group (TB group). Plasmatic cytokine/chemokine/growth factor profiling was performed using Luminex technology. Tuberculosis severity was assessed by clinical and laboratory parameters. Participants from TB group (4.55%) and TB/Prex-SCoV-2 (0.00%) received the complete COVID-19 vaccination.

**Results:**

Among 35 participants with pulmonary TB, 22 were classified as TB/Prex-SCoV-2. The parameters associated with TB severity, together with hematologic and biochemical data were similar between the TB and TB/Prex-SCoV-2 groups. Among the signs and symptoms, fever and dyspnea were significantly more frequent in the TB group than the TB/Prex-SCoV-2 group (p < 0,05). A signature based on lower amount of plasma EGF, G-CSF, GM-CSF, IFN-α2, IL-12(p70), IL-13, IL-15, IL-17, IL-1β, IL-5, IL-7, and TNF-β was observed in the TB/Prex-SCoV-2 group. In contrast, MIP-1β was significantly higher in the TB/Prex-SCoV-2 group than the TB group.

**Conclusion:**

TB patients previously infected with SARS-CoV-2 had an immunomodulation that was associated with lower plasma concentrations of soluble factors associated with systemic inflammation. This signature was associated with a lower frequency of symptoms such as fever and dyspnea but did not reflect significant differences in TB severity parameters observed at baseline.

## 1 Introduction

Tuberculosis (TB) remains a significant global health concern, persisting as one of the deadliest infectious diseases worldwide even amid the COVID-19 pandemic. Brazil is the only country in the Americas that appears on the World Health Organization (WHO) list of priority countries (1) and in 2022, 81,604 new cases of TB were reported. Of these, 71,878 (88%) were diagnosed with pulmonary TB, and it led to the death of more than 5 thousand Brazilians (2.72 deaths per 100,000 inhabitants) (2).

COVID-19 is an infectious disease caused by SARS-CoV-2. As of April 7th, 2024, approximately 775,293,630 cases of COVID-19 have been reported worldwide, including 7,044, 637 deaths since January 2020 (3). In Brazil, from February 2020 to January 2024, 38,374, 307 cases and 708, 638 deaths were reported (4). In the first year of the pandemic, approximately 80% of COVID-19 patients experienced mild to moderate symptoms, while 20% presented severe disease that led to hospitalization or death (5).

Nevertheless, the COVID-19 pandemic has been significantly modified by vaccination, resulting in fewer severe cases and hospitalizations. In Brazil, the available vaccines have been AstraZeneca/Oxford (ChAdOx1, Covishield), Johnson & Johnson (Ad26.COV2.S), and Pfizer/BioNTech (BNT162b2, Comirnaty) - which are capable of stimulating a specific immune response to the spike protein of SARS-CoV-2 - and Sinovac (CoronaVac) - composed of total inactivated SARS-CoV-2 (6). Immunization with these vaccines has demonstrated good induction of cellular and humoral immune responses specific to SARS-CoV-2 antigens (7–9).

For both TB and COVID-19, severity can be associated with some conditions and comorbidities such as advanced age (10), being a Person Living with HIV/AIDS (PLWHA) (11), diabetes (12), hypertension, hepatitis, and chronic respiratory disease (13). Moreover, the Centers for Disease Control and Prevention has listed TB as a risk factor for severe forms of COVID-19 (14). The few studies evaluating aspects of SARS-CoV-2 infection in persons with TB have focused their efforts on severe cases of COVID-19, in hospitalized patients (15, 16). The acute viral illness usually has a short duration of approximately 2 weeks from the onset of symptoms and therefore, it poses a challenge for the identification of patients co-infected with TB and COVID-19 (17).

A study evaluating the immune response of participants with latent *Mycobacterium tuberculosis* (Mtb) infection (LTBI) demonstrated that previous SARS-CoV-2 infection results in immunomodulation with higher serum concentrations of cytokines such as IFN-γ, IL-2, TNF-α, IL-17A, GM-CSF, IL-1α, IL-1β, IL-6, IL-12, IL-4, IL-13, and lower concentrations of IL-5 and IL-10 compared to persons with LTBI not infected with SARS-CoV-2 (18). Another study of TB/COVID-19 co-infection showed higher plasma concentrations of TNF-α, MIP-1β, and IL-9 compared to COVID-19 patients, and higher concentrations of IL-1β, TNF-α, IL-17A, IL-5, FGF-basic, and GM-CSF compared to TB patients. They also described a reduction in cytokine production in response to SARS-CoV-2 or Mtb antigens (16). Using flow-cytometry, Riou and colleagues (2021) demonstrated that CD4^+^ T cells of patients with TB/COVID-19 had a decrease ability to respond specifically to SARS-CoV-2 or Mtb stimuli (15). In this context, although it has been suggested that patients with COVID-19 together with active TB or LTBI have immune response modulation (19), it is not clear whether mild SARS-CoV-2 infection interferes with the host systemic immune response, long term after the viral clearance, that could be associated with worst pulmonary disease prognosis.

During TB, some inflammatory markers have been studied as predictors of disease severity. This includes increased levels of C-reactive protein (CRP), elevated erythrocyte sedimentation rate (ESR), increased IL-6, IFN-γ, and augmented neutrophils and lymphocyte counts. TB can also alter circulating levels of other laboratory parameters such as platelet counts, albumin levels and liver enzymes (14, 20, 21). The interference of viral infections such as measles, influenza, and HIV in TB has been characterized by the induction of a downward immunomodulation that can last for months or years, and this interference may directly reflect on the (higher) incidence of TB (20–22).

The effect of previous infection with SARS-CoV-2 in TB cases is not yet fully explored. As inflammation is the immunopathological basis of both TB and COVID-19, the present work aimed to investigate if previous infection with SARS-CoV-2 impacts the systemic inflammatory profile normally observed in persons with TB, and the potential impact of this previous infection on clinical presentation and severity of TB disease.

## 2 Material and methods

### 2.1 Study design and setting

This was a observational cross-sectional study nested within a larger cohort of TB patients with or without a history of SARS-CoV-2 infection recruited in Laboratório de Pesquisa Clínica em Micobacterioses (LAPCLINTB), of the Instituto Nacional de Infectologia Evandro Chagas, FIOCRUZ, Brazil.

### 2.2 Participants and biologic samples

A total of 150 TB participants were included from July 29^th^ 2020 to May 22^nd^ 2023. They were recruited in the LAPCLINTB outpatient clinic and the Hospital Center COVID-19 in the National Institute for Infectious Diseases Evandro Chagas, Fiocruz, Rio de Janeiro - Brazil for the Associative BRICS Research in COVID-19 and Tuberculosis (ABRICOT) study. To be eligible for the ABRICOT study, participants had to sign the informed consent form (ICF); be at least 18 years-old; had confirmed pulmonary TB. The exclusion criteria included treatment for >1 week for the current episode of TB; pregnancy or lactation; having a diagnosis of drug resistant (DR)-TB.

For the present study, in addition to the ABRICOT general inclusion/exclusion criteria, other selection criteria were considered: TB group - persons with TB and without previous infection with SARS-CoV-2; and TB/Prex-SCoV-2 group - persons with TB and previous infection with SARS-CoV-2. The definition of these two groups was based on vaccination history and a serological test specific for SARS CoV-2. All patients in the TB group had 3 negative serological tests (IgG anti-S1; IgA anti S1; and IgG anti-NCP). Patients in the TB/Prex-SCoV-2 group included those with no history of vaccination for COVID-19 and at least 1 of 3 positive serological tests for SARS-CoV-2; and patients with a history of vaccination with AstraZeneca/Oxford (ChAdOx1, Covishield), Johnson & Johnson (Ad26.COV2.S), or Pfizer/BioNTech (BNT162b2, Comirnaty), combined with positive IgG specific to SARS-CoV-2 nucleocapsid antigen. Since the patients in the TB group were all from the LAPCLINTB outpatient clinic, we selected participants in the TB/Prex-SCoV-2 from the outpatient clinic to have a similar patient population. We also excluded the following patients: 12 persons PLWHA to minimize the interference of immune dysregulation caused by chronic HIV infection; 7 patients who did not have clear information on vaccination for COVID-19; 5 patients who did not have plasma samples available in a biorepository; 40 patients who received the Sinovac (CoronaVac) COVID-19 vaccine as it induces an immune response to several SARS-CoV-2 antigens, hence serology results were insufficient to differentiate the vaccine-induced immune response from the immune response related to natural SARS-CoV-2 infection. In the end, 13 participants from the TB group and 22 participants from the TB/Prex-SCoV-2 group, recruited from July 2020 to July 2022, were analyzed.

All of these participants from the outpatient clinic were not subjected to specific RNA tests and/or SARS-CoV-2 antigens due to lack of signs and symptoms of COVID-19 checked a day before the scheduled visit.

Plasma from blood collected with EDTA tube were obtained at the baseline visit of the enrolled participants and stored at -80°C until utilization. The plasma samples were analyzed by Luminex assay to identify an immunologic signature in TB and TB/Prex-SCoV-2 participants, and by serological tests to assess for the presence of anti-SARS-CoV-2 antibodies.

### 2.3 Variables and definitions

Demographic variables such as age and sex were compared. Signs and symptoms, such as presence of cough, cough duration, fever, fatigue, night sweats, weight loss >10%, body mass index (BMI), chest pain, headache, lymphadenopathy, dyspnea, anorexia/hyperoxia, sputum, hemoptysis, odynophagia, dysphagia, hoarseness and Karnofsky score (23, 24) were also assessed at the baseline visit.

During the baseline visit, patients answered a questionnaire about their previous history of COVID-19 that included questions such as: 1. How long did they have COVID-19; 2. Was hospitalization required; 3. Was a laboratory-confirmed COVID-19 test available; and 4. Were any doses of vaccine against COVID-19 taken. COVID-19 vaccination was considered a complete cycle when at least three doses of any of the available vaccines in Brazil were administered. An incomplete cycle was defined as when there was at least one missing dose; unvaccinated was defined as no vaccine doses taken.

Pulmonary TB was defined as a positive sputum Xpert-MTB-RIF ULTRA and/or positive culture with identification of Mtb. Sputum smear grade was analyzed for bacillary load quantified by acid-fast bacilli (AFB) smear; Variables selected to explore TB severity were: BMI; Karnofsky score; disseminated TB (i.e., pulmonary and extrapulmonary TB clinical forms concomitantly); cavitation on chest X-ray; age; and weight loss >10%. Hematologic parameters (hematocrit, hemoglobin, white blood count, absolute neutrophil count, myelocytes, metamyelocytes, segmented neutrophils, banded neutrophils, neutrophil percentage, absolute lymphocyte count, lymphocyte percentage, platelets) and biochemical parameters (alanine aminotransferase, aspartate aminotransferase, gamma glutamyl transferase, alkaline phosphatase, albumin, total bilirubin, conjugated direct bilirubin, unconjugated indirect bilirubin, creatinine, urea, uric acid, CRP, lactate dehydrogenase) were also evaluated.

The comorbidities assessed were diabetes mellitus (defined as glycated hemoglobin >6.5%; prediabetes was defined as 5.7–6.4%), and chronic obstructive pulmonary disease (COPD)/emphysema, hypertension, and bronchitis (self-reported), that were grouped as the “other comorbidities” variable.

Previous exposure to SARS-CoV-2 was defined by: participants with no history of immunization for COVID-19 who had any positive serological test for anti-Spike (S1) IgA, anti-S1 IgG, or anti-Nucleocapsid (NCP) IgG at baseline; those participants previously vaccinated with AstraZeneca/Oxford (ChAdOx1, Covishield), Johnson & Johnson (Ad26.COV2.S), or Pfizer/BioNTech (BNT162b2, Comirnaty) vaccines (which stimulate an immune response specific to S protein), that had a positive test for IgG anti-NCP at baseline (7, 25).

### 2.4 Luminex assay

The cytokine/chemokine/growth factor profiling was performed utilizing the MILLIPLEX MAP Human Cytokine/Chemokine - Premixed 29 Plex (Merck, Hesse, Germany). This comprehensive panel allowed for the simultaneous quantification of the following analytes: EGF, Eotaxin, G-CSF, GM-CSF, IFN-α2, IFN-γ, IL-10, IL-12(p40), IL-12(p70), IL-13, IL-15, IL-17, IL-1RA, IL-1α, IL-1β, IL-2, IL-3, IL-4, IL-5, IL-6, IL-7, IL-8, IP-10, MCP-1, MIP-1α, MIP-1β, TNF-α, TNF-β, and VEGF. Measurements were taken in accordance with the manufacturer’s instructions. The Luminex Intelliflex xMAP technology was utilized for the detection and quantification of these soluble factor concentrations (pg/mL). The assay sensitivity (minimum detectable concentrations, pg/mL) of each analyte is represented in **Supplementary Table S2**.

### 2.5 Anti-SARS-CoV-2 antibody detection

Specific antibody against SARS-CoV-2 antigens were detected by enzyme linked immunosorbent assay in accordance with manufacture instructions (Euroimmun, Lubeck, Germany). Briefly, previously sensitized 96 well microplates with S1 domain from the spike protein (for detection of IgG or IgA) or NCP (for detection of IgG) were incubated with plasma samples diluted 1:100, together with negative control, positive control, and calibrator. The final result was expressed by the ratio obtained with the division of optical density of the patient’s plasma sample per the optical density from calibrator sample. The ratio <0.8 was considered a negative result; ratio ≥0.8 and <1.1 was considered indeterminate; and ratio ≥1.1 was considered a positive result. The assay’s sensitivity and specificity for each test were described as: anti S1 IgA (sensitivity: 87,5%; specificity:98.3%); anti S1 IgG (sensitivity: 94.4%; specificity:99.6%); and anti NCP IgG (sensitivity: 94.6%; specificity:99.8%).

### 2.6 Data analysis

Descriptive statistics were used to present data, and median values with interquartile ranges (IQR) were used as measures of central tendency and dispersion, for continuous variables. Categorical variables were described using frequency (n) and proportions (%). The chi-square test was used to compare categorical variables between study groups. The Mann-Whitney U test (for two unmatched groups) was used to compare continuous variables. To evaluate the overall profile of inflammation, the soluble factor data were converted to Log10 and an unsupervised hierarchical cluster analysis (Ward’s method), with dendrograms representing the Euclidean distances was performed. Log10 fold-changes were also calculated. The Spearman rank test was used to assess correlated biomarkers in each group. In all analyses, differences with p-values < 0.05 were considered statistically significant. The statistical analyses were performed using R (version 4·4·1).

## 3 Results

**Figure 1:**
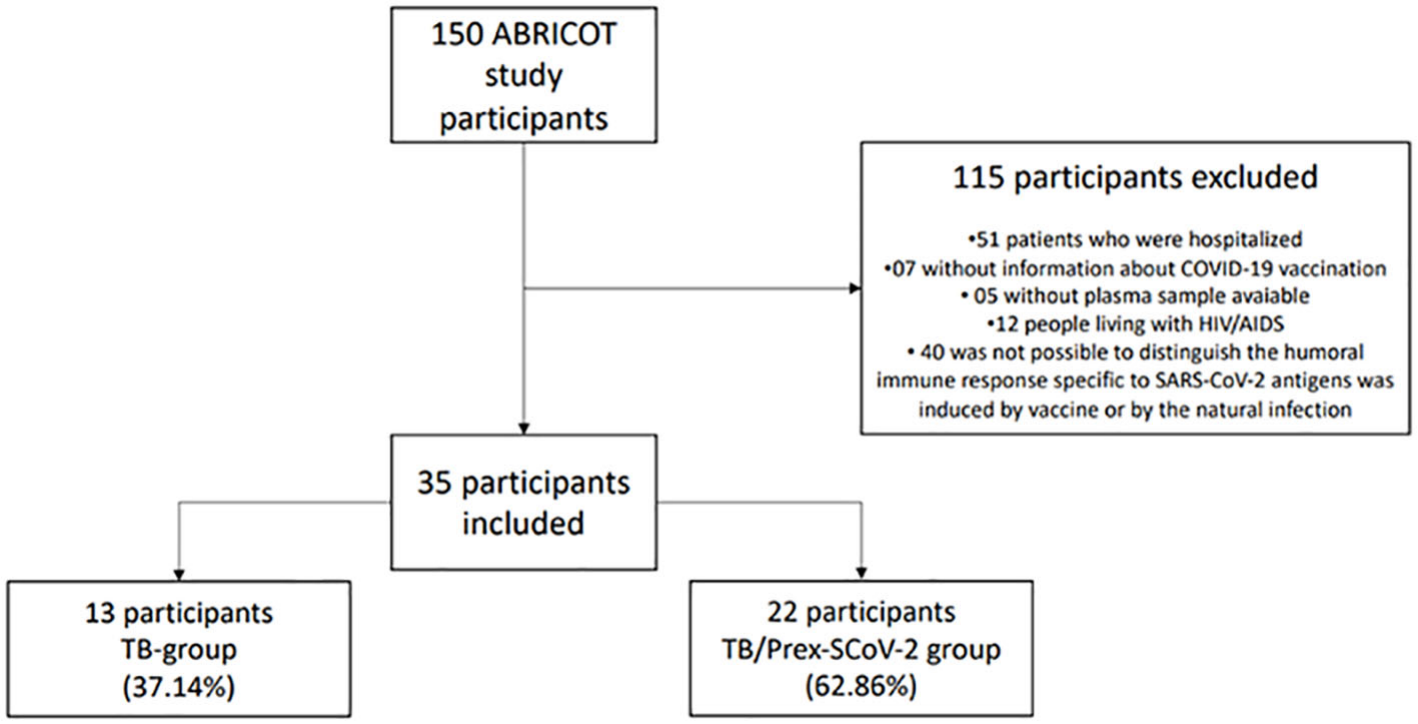
Sample selection scheme and the reasons that led to non-inclusion of participants for analysis.

We included 35 participants: 13 (37.1%) in the TB group and 22 (62.9%) in TB/Prex-SCoV-2 group (**Figure 1**). At the baseline visit 12 (92.3%) of the TB group and 13 (59.1%) of the TB/Prex-SCoV-2 group were not vaccinated, while no one and one subject (4.5%) were completely vaccinated, in the respective groups (**Table 1**). No significant difference was seen between the groups in relation to the frequency of comorbidities such as, diabetes mellitus, COPD, hypertension, and other less frequent conditions. Comparison of both groups for TB severity also displayed no differences. TB bacillary load was also similar between the groups (**Table 1**). Regarding hematologic and biochemistry parameters, both groups had lymphopenia and an increase in CRP, but the differences were insignificant (**Table 2**). Among the signs and symptoms analyzed, fever and dyspnea were significantly more frequent in the TB group than the TB/Prex-SCoV-2 (p<0.05) (**Table 3**).

**Table 1:** Demographic and clinical characteristics of tuberculosis (TB) and TB patients previously infected with SARS-CoV-2 (TB/Prex-SCov-2) at the baseline visit.

	TB (n=13)	TB/Prex-SCoV-2 (n=22)	p-value
Age, years (Median) [IQR 25% - 75%]	32 [24.2;40.0]	43 [30.5;59.5]	0.267
Male (%)	9 (69.2%)	14 (63.6%)	1.000
Body Mass Index (Median) [IQR 25% - 75%]	19 [18.3;21.4]	22 [17.3;23.9]	0.609
Weight loss >10% (%)	6 (46.2%)	8 (36.4%)	0.830
Karnofsky Score (Median) [IQR 25% - 75%]	0.8 [0.80;0.90]	0.9 [0.8;0.9]	0.761
Dysglycemia			0.379
Diabetes (%)	1 (7.69%)	6 (27.3%)	
Prediabetes (%)	4 (30.8%)	6 (27.3%)	
Missing HbA1c data (%)	7 (53.8%)	2 (9.09%)	
Other comorbidities*	3 (23.1%)	5 (22.7%)	1.000
Previous TB	2 (15.4%)	4 (18.2%)	0.604
TB clinical form			0.371
Pulmonary (%)	12 (92.3%)	22 (100.0%)	
Pulmonary and extrapulmonary (%)	1 (7.69%)	0 (0.00%)	
X Ray – cavitation (%)	8 (61.5%)	12 (54.5%)	1.000
Missing X Ray data (%)	1 (7.69%)	2 (9.09%)	
Sputum AFB load			0.274
1 to 9 bacili (%)	3 (27.2%)	1 (4.7%)	
1+ (%)	2 (18.2%)	4 (19.0%)	
2+ (few) (%)	2 (18.2%)	6 (28.6%)	
3+ (many) (%)	2 (18.2%)	4 (19.0%)	
Missing AFB data (%)	2 (18.2%)	1 (4.7%)	
COVID-19 vaccination status at baseline			0.109
Not vaccinated (%)	12 (92.3%)	13 (59.1%)	
Complete vaccination (%)	0 (0.00%)	1 (4.55%)	
Incomplete vaccination (%)	1 (7.69%)	8 (36.4%)	
History of COVID-19	0 (0.00%)	4 (18.0%)	0.273
How long had COVID-19 (days) Median [IQR 25% - 75%]	n.a.	378 [230.5;498.5]	n.a.
IgG anti-S1 (Ratio median) [IQR 25% - 75%]	0.12 [0.10;0.15]	4.13 [2.22;7.80]	<0.0001
IgA anti-S1 (Ratio median) [IQR 25% - 75%]	0.23 [0.16;0.35]	5.73 [2.19;11.03]	<0.0001
IgG anti-NCP (Ratio median) [IQR 25% - 75%]	0.19 [0.10;0.50]	1.76 [0.55;4.83]	0.0009

HbA1c – glycated hemoglobin, values >6.5 was considered as diabetes, and between 5.7- 6.4 was pre-diabetes; *Other comorbidities: chronic obstructive pulmonary disease (COPD)/Emphysema, hypertension, hepatitis B virus (HBV), and bronchitis. n.a: not applicable. Statistically significant levels (p<0.05) were highlighted in bold.

**Table 2:** Hematologic and biochemistry parameters for participants in the tuberculosis (TB) and previous infection with SARS-CoV-2 (TB/Prex-SCoV-2) groups at the baseline visit.

	TB (n=13) median [IQR]	TB/Prex-SCoV-2 (n=22) median [IQR]	p-value
Hematocrit, %	39.3 [35.9;41.7]	35.3 [32.6;41.7]	0.239
Hemoglobin, g/dL	12.7 [11.9;13.6]	11.4 [10.6;13.6]	0.260
White Blood Count, 10^3^/uL	10,190 [8,100;10,810]	9,770 [8,518;13,615]	0.720
Absolute neutrophil count, 10^3^/uL	7,983 [5,266;9,783]	7,334 [4,644;10,033]	0.682
Myelocytes, 10^3^/uL	0.0 [0.00;0.00]	0.0 [0.00;0.00]	0.442
Metamyelocytes, 10^3^/uL	0.0 [0.00;0.00]	0.0 [0.00;0.00]	0.835
Segmented neutrophils, 10^3^/uL	6,919 [5,030;9,375]	7,324 [5,331;10,029]	0.946
Banded neutrophils, 10^3^/uL	236,0 [103,0;408.0]	148.0 [80.2;273.0]	0.402
Neutrophil, %	80.0 [67.0;90.0]	72.5 [63.0;80.0]	0.422
Absolute lymphocyte count, 10^3^/uL	1,495 [1,081;1,674]	1,701 [1,296;2,753]	0.142
Lymphocyte, %	14.0 [8.00;22.0]	18.5 [13.0;27.8]	0.200
Platelets, mm^3^	426,000 [392,000;486,000]	448,000 [290,500;614,250]	0.946
Alanine aminotransferase (ALT), U/L	25.0 [20.0;32.0]	19.5 [16.2;29.8]	0.297
Aspartate aminotransferase (AST), U/L	29.0 [24.0;43.0]	25.5 [20.0;33.8]	0.274
Gamma glutamyl transferase (GGT), U/L	74.0 [42.0;109.0]	83.0 [46.2;124.0]	0.878
Alkaline Phosphatase (ALP), U/L	91.0 [67.0;130.0]	110.0 [95.2;134.0]	0.167
Albumin. g/dL	2.9 [2.81;3.53]	3.2 [2.76;3.70]	0.696
Total Bilirubin, mg/dL	0.44 [0.31;0.49]	0.38 [0.26;0.59]	1.000
Conjugated direct bilirubin, mg/dL	0.13 [0.09;0.24]	0.10 [0.09;0.18]	0.705
Unconjugated indirect bilirubin, mg/dL	0.29 [0.18;0.32]	0.24 [0.16;0.42]	0.905
Creatinine, mg/dL	0.91 [0.79;1.08]	0.81 [0.69;0.87]	0.133
Urea, mg/dL	23.0 [19.0;29.0]	26.0 [18.0;34.0]	0.750
Uric acid, mg/dL	4.70 [3.80;5.40]	4.25 [3.48;5.43]	0.606
C-reactive protein (CRP), mg/dL	6.72 [3.14;10.50]	6.91 [2.56;9.47]	0.575
Lactate Dehydrogenase (LDH), U/L	171 [157;247]	167 [165;210]	0.841

**Table 3:** Signs and symptoms registered at the baseline visit of out clinic tuberculosis patients (TB) and previous infection with SARS-CoV-2 (TB/Prex-ScoV-2).

Signs and Symptoms	TB (n=13)	TB/Exp-SCoV-2 (n=22)	p value
Cough (%)	13 (100.0%)	20 (90. 9%)	0.519
Cough duration (weeks) Median (IQR 25% - 75%)	10 (3.0; 30.5)	12 (5.0; 25.5)	0.591
Fever (%)	11 (84.6%)	10 (45.4%)	**0.034**
Weight loss (%)	11 (84.6%)	17 (77.3%)	0.689
Fatigue (%)	10 (76.9%)	16 (72.3%)	1.000
Chest pain (%)	9 (69.2%)	13 (61.9%)	0.727
Night sweats (%)	12 (92.3%)	13 (59.1%)	0.055
Headache (%)	2 (15.4%)	4 (18.2%)	1.000
Adenomegaly (%)	0 (0.0%)	2 (9.1%)	0.519
Anorexia/hiporexia (%)	8 (61.5%)	14 (63.6%)	1.000
Dyspnea (%)	11 (84.6%)	9 (40.9%)	**0.016**
Sputum (%)	6 (46.1%)	14 (63.6%)	0.551
Hemoptysis (%)	2 (15.4%)	5 (22.7%)	0.689
Odynofagy (%)	0 (0.0%)	0 (0.0%)	1.000
Hoarseness (%)	1 (7.7%)	0 (0.0%)	0.371

Statistically significant levels (p<0.05) were highlighted in bold.

Although it was not possible to precisely specify the time of previous SARS-CoV-2 infection in the TB/Prex-SCoV-2 group, 4/22 (18%) of patients reported a previous history of COVID-19, with 3 of the 4 with a confirmed PCR or antigen test. Based on the dates provided by the participants of their COVID-19 infection, symptomatic infection occurred a median of 378 days before then visit, with the longest time of 503 days and the shortest of 217 days before the visit. Most individuals (82%) in the TB/Prex-SCoV-2 group had few or no COVID-19-related symptoms. The results of specific serology for SARS-CoV-2 antigens showed that the TB group did not have reactivity to the different tests, while the TB/Prex-SCoV-2 group had significantly higher concentrations of specific antibodies (**Supplementary Figure S1**).

**Figure 2:**
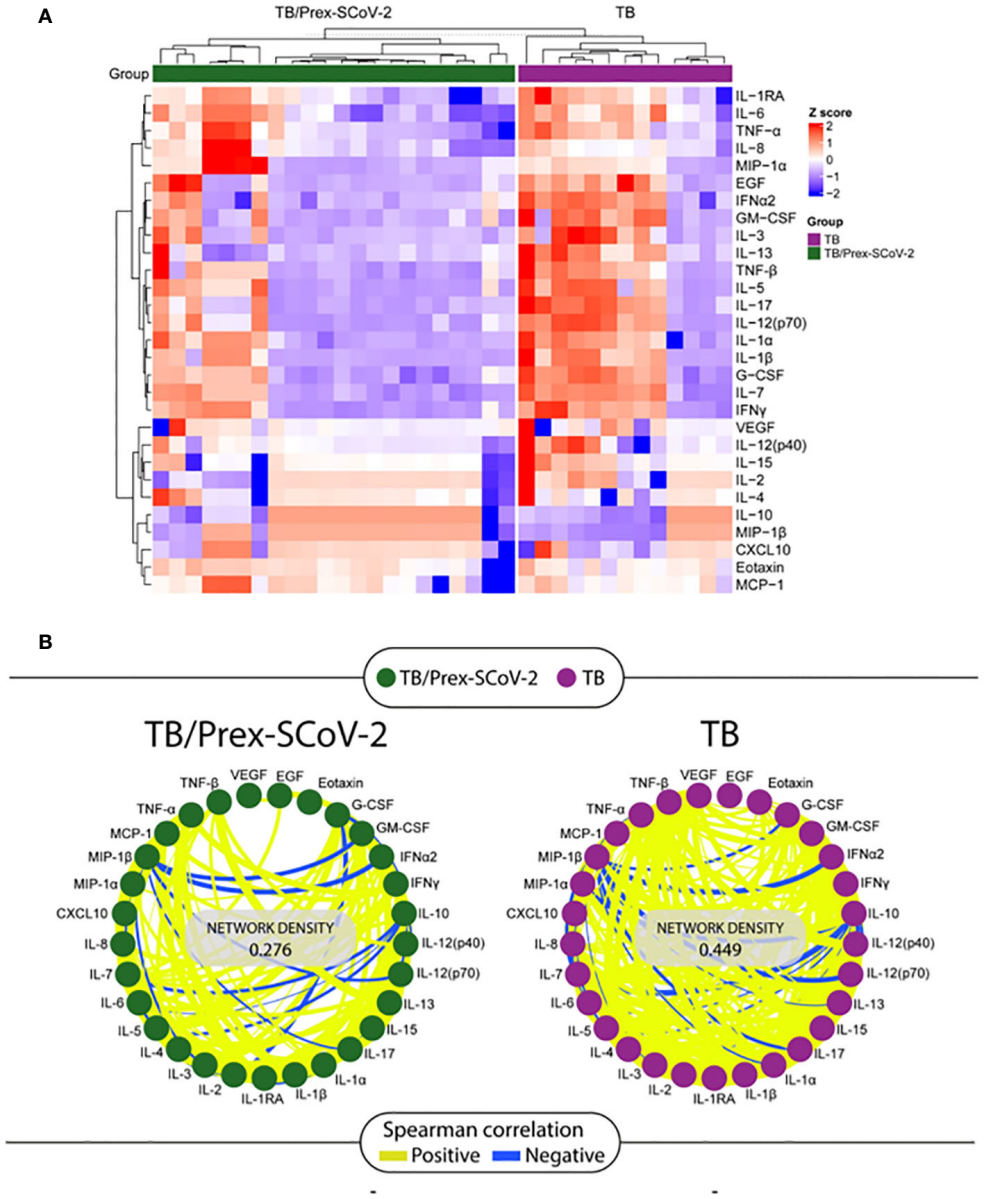
Analysis of the soluble factors quantified by the Luminex assay in plasma samples obtained at the baseline visit from participants in the TB and the TB/Prex-SCoV-2 groups. **(A)** Heat map with a hierarchical cluster analysis (Ward’s method, with dendrograms implying the Euclidean distance) was employed, and clustering defining the higher producers (in red) and lower producers (in blue) of inflammatory soluble factors in each group: TB/Prex-SCoV-2 in the upper horizontal green bar; and TB in the upper horizontal purple bar. **(B)** Correlation network of TB/Prex-SCoV-2 group (green nodes) and TB group (purple nodes) at the baseline visit. The connecting lines represent statistically significant correlations (p < 0.05). Yellow connecting lines represent positive correlations while blue lines infer negative correlations. The thickness of the line is directly related to the strength of the correlation. The network density values were obtained based on the strength and the number of positive or negative correlations observed for each group.

The Luminex analysis of plasma samples showed an inflammatory signature with a significantly lower concentration of soluble factors such as EGF, G-CSF, GM-CSF, IFN-α2, IL-12(p70), IL-13, IL-15, IL-17, IL-1β, IL-5, IL-7, and TNF-β and a significantly higher MIP-1β for TB/Prex-SCoV-2 group in relation to the TB group (**Table 4**). The data show a clear clustering of certain soluble factors in both groups that defined patients with high production (red color means high concentration) and low production (blue color means low concentration) of soluble factors such as IL-1RA, IL-6, TNF-α, IL-8, MIP-1α, EGF, IFN-α2, GM-CSF, IL-3, IL-13, TNF-β, IL-5, IL-17, IL-12(p70), IL-1α, IL-1β, G-CSF, IL-7, and IFN-γ (**Figure 2A**). The frequency of the low producers’ profile of inflammatory factors in the TB/Prex-SCoV-2 group (65.0%) was significantly higher than in the TB group (30.7%) (p<0.05). A lower density of Spearman’s correlations was observed for the TB/Prex-SCoV-2 group when compared to the TB group (**Figure 2B**).

**Table 4:** Inflammatory plasmatic soluble factors of tuberculosis (TB) patients and TB patients previous infected with SARS-CoV-2 (TB/Prex-ScoV-2) at the baseline visit.

Soluble factors (pg/ml)	TB (n=13) median [IQR]	TB/Prex-SCoV-2 (n=22) median [IQR]	p-value
EGF	23.40 [1.87;74.6]	0.70 [0.46;1.58]	**0.015**
Eotaxin	93.8 [77.6;101,0]	108.0 [86.8;116.0]	0.219
G-CSF	141.0 [12.2;259.0]	13.0 [7.81;79.4]	**0.012**
GM-CSF	11.80 [0.53;28.70]	0.56 [0.32;1.87]	**0.025**
IFN-α2	193.0 [35.3;273.0]	28.2 [24.3;42.5]	**0.006**
IFN-γ	30.80 [0.93;34.00]	1.08 [0.70;18.10]	0.101
IL-10	14.3 [7.6;182.0]	182.0 [19.8;183.0]	0.109
IL-12(p40)	9.56 [8.71;21.30]	8.99 [8.78;10.50]	0.463
IL-12(p70)	6.85 [0.47;11.90]	0.37 [0.24;1.58]	**0.004**
IL-13	7.82 [2.70;10.20]	2.32 [1.85;2.70]	**0.001**
IL-15	13.5 [13.2;18.2]	13.0 [12.6;13.3]	**0.034**
IL-17	11.70 [1.59;22.10]	0.62 [0.28;1.06]	**0.003**
IL-1RA	39.40 [16.30;53.70]	15.40 [9.19;28.40]	0.060
IL-1α	23.60 [4.80;35.9]	4.96 [4.20;24.1]	0.172
IL-1β	1.52 [0.08;3.61]	0.07 [0.05;0.78]	**0.019**
IL-2	8.94 [8.50;12.1]	8.86 [3.21;8.92]	0.076
IL-3	1.23 [0.19;3.06]	0.20 [0.11;0.27]	0.065
IL-4	88.0 [69.0;105]	102.0 [73.7;103.0]	0.864
IL-5	3.47 [0.33;8.81]	0.14 [0.10;2.19]	**0.029**
IL-6	9.63 [6.47;22.90]	3.49 [0.63;15.90]	0.076
IL-7	18.50 [1.74;23.40]	0.85 [0.56;10.00]	**0.026**
IL-8	9.28 [6.60;15.90]	6.56 [3.15;16.70]	0.260
IP-10	956 [480;2,373]	2,317 [1,084;2,513]	0.306
MCP-1	164 [137;253]	180 [115;330]	0.759
MIP-1α	13.70 [0.64;19.50]	1.00 [0.27;15.60]	0.282
MIP-1β	22.8 [16.1;356.0]	360.0 [113.0;361.0]	**0.024**
TNF-α	15.80 [7.89;22.70]	8.11 [5.74;11.90]	0.142
TNF-β	14.6 [1.49;36.80]	1.02 [0.73;10.70]	**0.013**
VEGF	102.0 [98.9;132.0]	102.0 [99.6;121.0]	0.759

Statistically significant levels (p<0.05) were highlighted in bold.

## 4 Discussion

The TB and TB/Prex-SCoV-2 groups were similar in terms of demographic characteristics, comorbidities, laboratory findings, COVID-19 vaccination status, and TB severity. The low COVID-19 vaccination rate observed in both groups can be explained by the younger age of participants; the elderly population had priority in the vaccination campaign in Brazil. Although the groups were comparable in terms of baseline characteristics and severity of cases, fever and dyspnea were more frequent in the TB group. The lower frequency of dyspnea in the TB/Prex-SCoV-2 group may have been a residual effect of SARS-CoV-2 infection. It is likely that the higher frequencies of symptoms (fever and dyspnea) observed in the present study may be related to the higher systemic concentration of inflammatory soluble factors observed in the TB group.

The signature defined by lower concentrations of plasma soluble factors was observed in the TB/Prex-SCoV-2 group. The present work suggests that previous SARS-CoV-2 infection interferes with the systemic immunity usually observed in TB patients. This immunological disturbance, probably triggered by previous exposure to SARS-CoV-2, was supported by the low network density in the Spearman correlation matrix. The supposed immune modulation occurring after elimination of the virus, suggests that the remaining SARS-Cov-2 stimuli persisted long term within the host after virus clearance (26, 27). This persistent specific immunity was also observed for patients with mild and moderate COVID-19 disease (28). In contrast to our observations, Rajamanickam and colleagues (2022) showed that in individuals with LTBI there was systemic immunomodulation when previously infection with SARS-CoV-2, with a higher circulation of cytokines/chemokines, associated with Mtb control (18).

Our findings showed that the TB/Prex-SCoV-2 group had lower amounts of systemic soluble factors such GM-CSF (29), IL-15 (30), IL-1β (31), IL-7 (30), TNF-β (32), IL-12(p70) (33), IL-17 all of which are considered essential for Mtb control (34). Nevertheless, there was no difference in the bacillary load in sputum when compared to the TB group. In addition, lower concentrations of IL-5, IL-13 (35) and IFN-α2 (36) (involved in the induction of an immune response associated with favoring Mtb infection) were not correlated with increased bacillary load. So, the immunomodulation caused by previous SARS-CoV-2 infection did not seem to have a reflex in hematological or biochemical laboratory results. We expected to see more severe cases of TB in the TB/Prex-SCoV-2 group due to the lower levels of Th1 and inflammatory soluble factors, but this low production profile did not seem to affect TB severity or inflammation (other inflammatory markers such as RCP were also similar between the two groups). However, fever and dyspnea were significantly more frequent in the TB group, demonstrating a difference in clinical findings in the TB/Prex-SCoV-2 group, probably due to the higher amounts of blood inflammatory factors observed in the TB group.

None of the clinical, demographic, or laboratory parameters could be associated with the low amount of plasma soluble factor, which was more frequently observed in the TB/Prex-SCoV-2 group. In a complementary analysis within the TB/Prex-SCoV-2 group, we did not find a systemic immune response profile associated with the type of vaccine (AstraZeneca or Pfizer) or the number of doses (1 or 2) received in relation to unvaccinated individuals. So, vaccination for COVID-19 did not interfere with the systemic immune response profile, even though it was at a low frequency among individuals in the TB group. it is important to note that these patients were included in the study from July 2020 to July 2022, a period in which several variants of SARS-CoV-2 circulated in Brazil, with a direct impact on the host’s immune response (37, 38).

In conclusion, TB patients previously infected with SARS-CoV-2 demonstrated an immunomodulation that conferred lower plasma concentrations of growth factor (EGF), innate immunity markers (G-CSF, GM-CSF and IFN-α2), Th1 profile (IL-12[p70] and IL-15), Th17 profile (IL-17), Th2 profile (IL-5 and IL-13), inflammatory cytokines (TNF-β and IL-1β), and memory lymphocytes associated factor (IL-7). This disturbance in systemic immunity was associated with a lower frequency of symptoms such as fever and dyspnea in the TB/Prex-ScoV-2 group, but was not associated with significant differences in the severity of TB, hemogram and biochemistry parameters at the baseline visit compared to persons with only TB.

## 5 Limitations

Selection of participants was based on TB diagnosis with Xpert-MTB-RIF or Mtb culture. INI is a tertiary hospital of infectious diseases and maybe for this reason comorbidities could have been more frequent than in basic health clinics. Diagnosis of previous SARS-CoV-2 infection based on serology has limitations, due to cross reactivity with antibodies induced by the COVID-19 vaccine, or inability of the immune system to produce enough specific antibodies detectable by available serological methods, especially long after exposure. However, all efforts were made to select vaccinated participants based on the specificity of the immune response, i.e., patients vaccinated with S immunogen might have presumed natural infection when they presented specific antibodies to NCP antigen. This difficulty had a direct impact on the small sample size in each group, but it is important to note the selected participants were carefully classified according to their previous infection or not with SARS-CoV-2. It was not possible to specify the time of previous SARS-CoV-2 infection in the TB/Prex-SCoV-2 group, and to measure if these patients were infected more than once. Therefore, most of the patients selected for this study were unvaccinated individuals, so a relationship between vaccination for COVID-19 and its interference in the TB systemic immune response profile could not be established.

SARS-CoV-2 variants infecting the TB/Prex-SCoV-2 participants were not characterized, and because participants were included from July 29^th^ 2020 to July 05^st^ 2022 several SARS-CoV-2 lineages were potentially involved which could directly impact on the activation of distinct immune response profiles.

## Data availability statement

The original contributions presented in the study are included in the article/**Supplementary Materials**. Further inquiries can be directed to the corresponding authors.

## Ethics statement

The studies involving humans were approved by “Comitê de ética em pesquisa em humanos do Instituto Nacional de Infectologia Evandro Chagas - INI/FIOCRUZ”. The studies were conducted in accordance with the local legislation and institutional requirements. The participants provided their written informed consent to participate in this study. Written informed consent was obtained from the individual(s) for the publication of any potentially identifiable images or data included in this article.

## Author contributions

MX: Data curation, Investigation, Writing – review & editing. MA-P: Data curation, Formal analysis, Validation, Visualization, Writing – review & editing. QD: Investigation, Methodology, Project administration, Supervision, Writing – review & editing. FS: Investigation, Methodology, Writing – review & editing. FR: Methodology, Writing – review & editing. AD: Methodology, Writing – review & editing. MF: Supervision, Writing – review & editing. TS: Supervision, Writing – review & editing. BG: Supervision, Writing – review & editing. BK: Supervision, Writing – review & editing. BA: Data curation, Formal analysis, Methodology, Writing – review & editing. VR: Conceptualization, Data curation, Formal analysis, Funding acquisition, Investigation, Project administration, Resources, Supervision, Writing – original draft, Writing – review & editing. AG-S: Conceptualization, Data curation, Formal analysis, Funding acquisition, Investigation, Methodology, Project administration, Resources, Supervision, Writing – original draft, Writing – review & editing.

## Supplementary material

Supplementary Figure 1 Level of plasma anti-S1 (IgA and IgG) and anti-NCP (IgG) antibodies. Each dot represents a participant. The horizontal bar indicates the median. The dotted lines define the range next to the cut off value (gray zone). The semi-quantitative results were expressed by the ratio, calculated between the absorbance of the samples and the absorbance of the calibrator. ****p<0.0001; ***p<0.001.

Supplementary Table 1 Excel file containing dataset of Spearman correlation results from Luminex soluble factors.

Supplementary Table 2 Minimum detectable concentrations specified by manufactured for each soluble factor dosage by Luminex assay.
