# Supplementary figures and images for: The effect of previous SARS-CoV-2 infection on systemic immune responses in individuals with tuberculosis
Source: Front Immunol. 2024 Jun 27;15:1357360. doi: 10.3389/fimmu.2024.1357360 (PMC11236595; doi:10.3389/fimmu.2024.1357360)

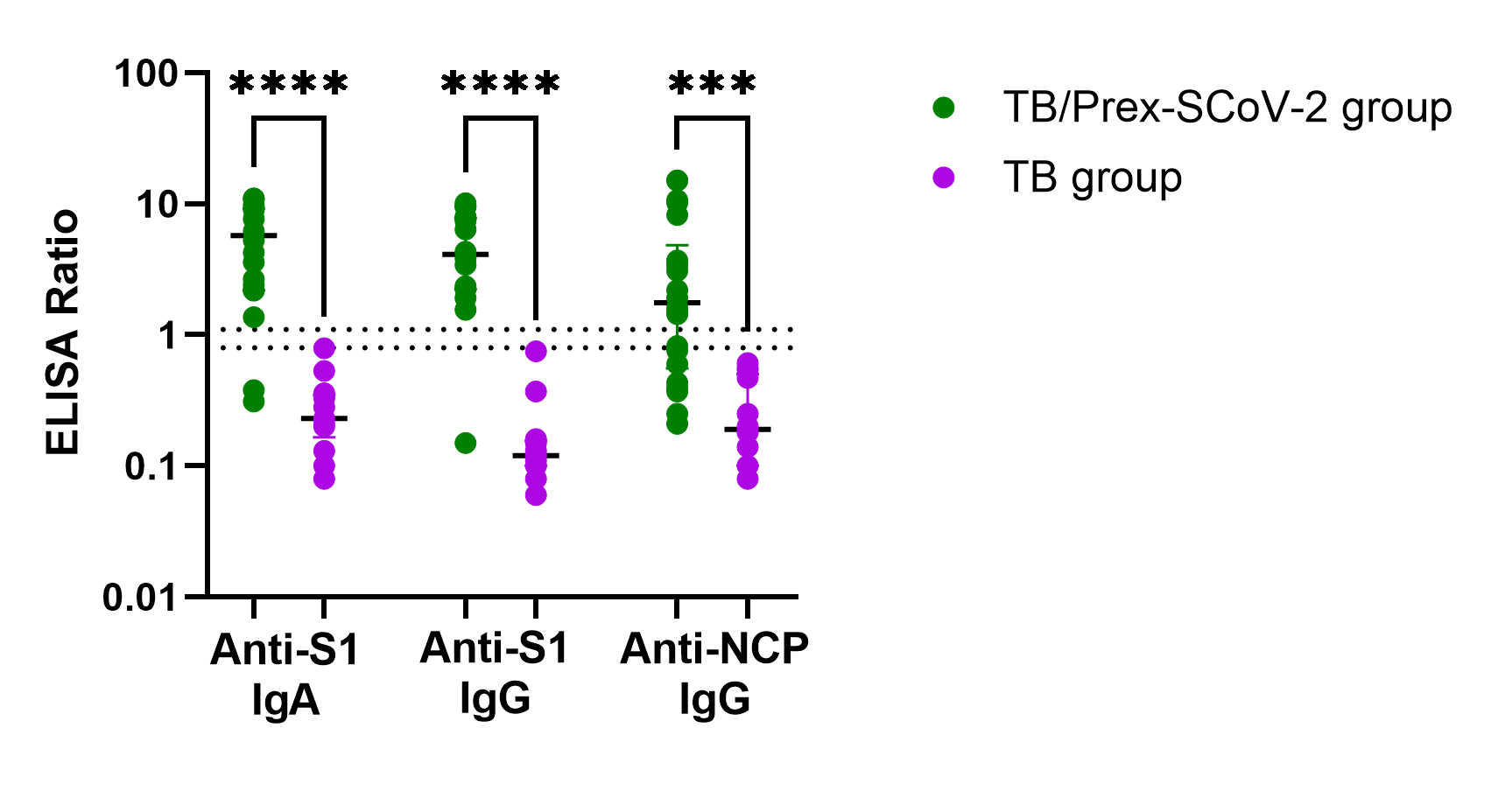

Supplement: Supplementary Figure 1 — Level of plasma anti-S1 (IgA and IgG) and anti-NCP (IgG) antibodies. Each dot represents a participant. The horizontal bar indicates the median. The dotted lines define the range next to the cut off value (gray zone). The semi-quantitative results were expressed by the ratio, calculated between the absorbance of the samples and the absorbance of the calibrator. ****p<0.0001; ***p<0.001. [file Image_1.tif]
