# Supplementary material for: The effect of previous SARS-CoV-2 infection on systemic immune responses in individuals with tuberculosis
Source: Front Immunol. 2024 Jun 27;15:1357360. doi: 10.3389/fimmu.2024.1357360 (PMC11236595; doi:10.3389/fimmu.2024.1357360)
Supplement: Supplementary Table 2 — Minimum detectable concentrations specified by manufactured for each soluble factor dosage by Luminex assay. [file Table_2.docx]

**Supplementary table 2**. Minimum detectable concentrations specified by manufactured for each soluble factor dosage by Luminex assay.

| **Soluble factors** | Minimum detectable concentrations (pg/mL) | Minimum detectable concentrations + (2 x SD) (pg/mL) |
| --- | --- | --- |
| EGF | 2.8 | 4.6 |
| Eotaxin | 4.0 | 6.8 |
| G-CSF | 1.8 | 3.3 |
| GM-CSF | 7.5 | 15.0 |
| IFN-a2 | 2.9 | 4.8 |
| IFN-y | 0.8 | 1.1 |
| IL-10 | 1.1 | 1.6 |
| IL-12P40 | 7.4 | 12.7 |
| IL-12P70 | 0.6 | 1.0 |
| IL-13 | 1.3 | 1.9 |
| IL-15 | 1.2 | 1.7 |
| IL-17 | 0.7 | 1.2 |
| IL-1RA | 8.3 | 17.1 |
| IL-1a | 9.4 | 12.6 |
| IL-1ß | 0.8 | 1.0 |
| IL-2 | 1.0 | 1.6 |
| IL-3 | 0.7 | 1.0 |
| IL-4 | 4.5 | 7.1 |
| IL-5 | 0.5 | 0.7 |
| IL-6 | 0.9 | 1.3 |
| IL-7 | 1.4 | 2.4 |
| IL-8 | 0.4 | 0.7 |
| IP-10 | 8.6 | 14.0 |
| MCP-1 | 1.9 | 3.4 |
| MIP-1a | 2.9 | 6.2 |
| MIP-1ß | 3.0 | 4.8 |
| TNFa | 0.7 | 1.1 |
| TNFß | 1.5 | 1.9 |
| VEGF | 26.3 | 47.9 |
